# Effect of Drought on Herbivore-Induced Plant Gene Expression: Population Comparison for Range Limit Inferences

**DOI:** 10.3390/plants5010013

**Published:** 2016-03-11

**Authors:** Gunbharpur Singh Gill, Riston Haugen, Steven L. Matzner, Abdelali Barakat, David H. Siemens

**Affiliations:** 1 Integrative Genomics Program, Black Hills State University, Spearfish, SD 57789, USA; Gunbharpur.Gill@yellowjackets.bhsu.edu (G.S.G.); Riston.Haugen@yellowjackets.bhsu.edu (R.H.); 2 Biology Department, Augustana University, Sioux Falls, SD 57197, USA; steven.matzner@augie.edu; 3 Biology Department, University of South Dakota, Vermillion, SD 57069, USA; Abdelali.Barakat@usd.edu

**Keywords:** drought, herbivory, glucosinolates, carbon isotope ratio, gene expression

## Abstract

Low elevation “trailing edge” range margin populations typically face increases in both abiotic and biotic stressors that may contribute to range limit development. We hypothesize that selection may act on ABA and JA signaling pathways for more stable expression needed for range expansion, but that antagonistic crosstalk prevents their simultaneous co-option. To test this hypothesis, we compared high and low elevation populations of *Boechera stricta* that have diverged with respect to constitutive levels of glucosinolate defenses and root:shoot ratios; neither population has high levels of both traits. If constraints imposed by antagonistic signaling underlie this divergence, one would predict that high constitutive levels of traits would coincide with lower plasticity. To test this prediction, we compared the genetically diverged populations in a double challenge drought-herbivory growth chamber experiment. Although a glucosinolate defense response to the generalist insect herbivore *Spodoptera exigua* was attenuated under drought conditions, the plastic defense response did not differ significantly between populations. Similarly, although several potential drought tolerance traits were measured, only stomatal aperture behavior, as measured by carbon isotope ratios, was less plastic as predicted in the high elevation population. However, RNAseq results on a small subset of plants indicated differential expression of relevant genes between populations as predicted. We suggest that the ambiguity in our results stems from a weaker link between the pathways and the functional traits compared to transcripts.

## 1. Introduction

The study of factors and processes affecting species range limits has a long history in ecology and evolution starting with Darwin, but there has been a recent resurgence of interest in range limits in part to understand some of the consequences of climate change [1,2,3,4]. Because most transplant experiments show poorer performance across range boundaries (Sexton *et al.* 2009 [2] for review), many range margin populations must face stressful environments that they are not adapted to. Therefore, understanding what prevents this adaptation may be key to understanding the development of range limits. Since there is often sufficient genetic variation for traits that matter within range margin populations, if there are also no barriers to dispersal, possible constraints on the process of adaptation include swamping gene flow from elsewhere in the range and tradeoffs. But because many range margin populations are also geographically and genetically isolated, it is thought that the study of range limit development should often focus on molecular, physiological or developmental tradeoffs [5]. What kind of tradeoffs might be constraining low elevation trailing edge populations?

At low latitudinal or altitudinal “trailing edge” range limits, populations are thought to more commonly face both abiotic and biotic stressors compared to high altitudes or latitudes at leading edges where abiotic stressors predominate [6]. Although there would be some exceptions to this pattern depending on latitude, altitude and local climate conditions, many cases should comply. For example, transplant experiments with the upland mustard species *Boechera stricta*, a close relative of *Arabidopsis*, resulted in lower survivorship across low elevation range boundaries that coincided with increased abiotic and biotic stressors such as decreased water availability and increased herbivory by generalist insect herbivores [7]. Presumably, increased drought tolerance and chemical defense levels would allow upland species like *B. stricta* to expand low elevation range boundaries. However, negative genetic correlations, *i.e.*, evolutionary tradeoffs, between glucosinolate (GS) toxin defense allocation and abiotic stress tolerances associated with low elevation range limits have been observed in *B. stricta* [7,8,9,10].

One hypothesis for the evolutionary tradeoff involves natural selection acting on antagonistic plastic response pathways [7,11]. The process of adaptation often proceeds by modifying existing structures and pathways. Within ranges, stress response signal transduction pathways help plants to survive temporary challenges from abiotic and biotic stressors [12]. Just across range boundaries, some of these same stressors increase in frequency; therefore, one would predict that adaptation to stressful environments across range limits would involve the up-regulation of stress response pathways such that the pathways and the traits that they regulate were expressed more frequently or stably. However, evolutionary models predict that a problem may arise when antagonistic response pathways are co-opted simultaneously for evolutionary change [13]. For example, from work on *Arabidopsis* it is well known that stress response pathways, such as Abscisic acid (ABA) signaling for coping with temporary challenges of abiotic stressors (e.g., drought), and Jasmonic acid (JA) signaling for coping with bouts of biotic stressors (e.g., herbivores) may negatively interfere with one another ([14,15,16] for reviews). Thus, the simultaneous co-option of these antagonistic pathways for low elevation range expansion where organisms face both increased abiotic and biotic stressors may be problematic because of the crosstalk.

The problem is with negative pleiotropic and epistatic effects that may constrain evolution. Multiple signaling pathways often form networks involving regulatory genes—transcription factors (TFs)—that may interact to produce multiple positive and negative integrative effects. If natural selection acts on genetic variation in either the coding regions or the *cis* regulatory regions of TFs involved in the networks, multiple pathways may be affected. Although some of the effects may be adaptive, many may be mal-adaptive. For example, the flowering time signaling network in *Arabidopsis* consists of many positive and negative interactions among photoperiod, circadian clock, vernalization, autonomous and Gibberellic acid pathways. Epistatic interactions between the major flowering time network genes FRI and FLC, were one of the contributing factors in the maintenance of genetic variation in *Arabidopsis* flowering time [17]. Thus, by preventing fixation of alleles, the epistasis represents an evolutionary constraint. FRI and FLC are major TFs in the flowering time signaling network that allow large behavioral shifts involving many genes, but these major effects might impede evolution.

It has also been shown that high and low elevation populations of *B. stricta* have diverged with respect to abiotic stress tolerance and glucosinolate defense levels as one would predict based on the above hypothesis; neither population had high values of both kinds of traits [11]. In a common garden growth chamber experiment, the two populations showed genetic divergence for glucosinolate content (*F*_18, 701_ = 7.101, *p* < 0.001) and stress tolerance traits such as root:shoot ratio (*F*_5, 251_ = 3.576, *p* = 0.004). The high elevation population showed higher inherent root:shoot ratios, while the lower elevation population was higher in glucosinolate levels. Further, in experimental crosses between the populations, the two kinds of traits would not segregate independently of one another in the F2 generation (Siemens *et al.*, unpublished data [18]: *F*_1, 599_ = 65.987, *p* < 0.001). Together, these results indicate a genetic tradeoff that probably involves negative pleiotropic or epistatic interactions.

Here, we compared the genetically diverged high and low elevation populations of *B. stricta* in a double challenge drought-herbivory growth chamber experiment to test the following predictions based on the central hypothesis that the evolutionary tradeoff derives from antagonistic plastic responses: (1) Drought stress inhibits herbivore-induced defense responses; (2) Induced abiotic stress responses are attenuated in the high elevation population that already shows high constitutive levels of the tolerance traits; (3) Likewise, herbivore-induced defense responses are attenuated in the low elevation population that already shows high basal levels of the defensive traits. In other words, predictions 2 and 3 state that high constitutive levels of traits would coincide with lower plasticity. Prediction 1 is just an expectation to determine whether antagonistic signaling exists.

## 2. Results

### 2.1. Flat Weights

**Figure 1:**
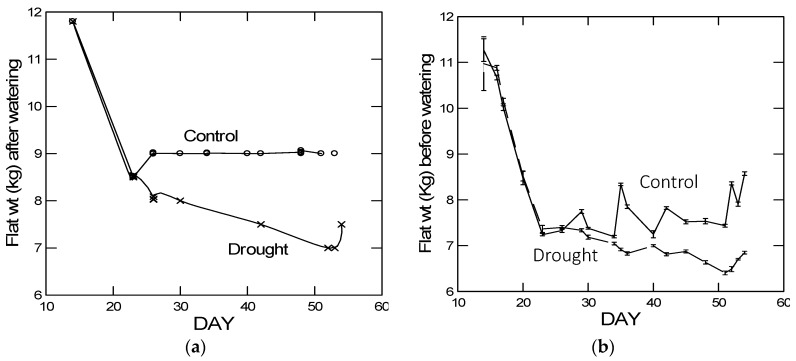
Flat weights (**a**) just after and (**b**) before or between watering for the control and drought treatments. All flats in each treatment were watered to the same weight, so there are no error bars for the after watering flat weights. For the before watering flat weights, error bars are ± 1SE across four flats for each watering treatment.

Drought treated flats were watered progressively with less amounts and less frequently than control treated flats over a 4-week period as documented by flat weights just after watering (Figure 1a). Between watering, the flat weights of the control watered flats fluctuated, but were always higher than those of the drought treated flats. By contrast, the flat weights of the drought treated plants remained low between watering treatments and steadily declined (Figure 1b). Eventually, on day 51 post-planting, 24 days after the drought treatments began, the flat weights of drought treated plants fell just below 6.5 kg. At this time, two plants in the drought treatment group had curled rosette leaves indicating early signs of wilting (Figure S1). Flat weights were then monitored more frequently and the flats were watered just enough (eventually to 7.5 kg, similar to flat weights on day 42 post-planting) to allow wilted plants to recover to previous non-wilted stress levels and to survive without further wilting, but still stressed, through the 2-day herbivore feeding treatments, which began four days later on day 55.

### 2.2. Betacyannin Color Score

By day 36, as water deficiency treatments progressed, the lower epidermis of drought treated plants began to turn a more violet red color (Betacyannin antioxidant stress response: *F*_1, 6_ = 15.1877, *p* = 0.008). This visual Betacyannin indicator of stress was the same for plants of both populations (no drought-by-population interaction: *F*_1, 235_ = 0.135, *p* = 0.713) (Table 1, Figure S2a).

### 2.3. Plant Growth Response

There was an eventual difference in the size of shoots between drought treatments (repeated measures within subjects ANOVA: time-by-drought interaction—*F*_4, 24_ = 6.281, *p* = 0.001) (Figure S2b). Although the populations differed in shoot growth (repeated measures between subjects ANOVA: *F*_4, 940_ = 6.946, *p* < 0.001), the effect of drought did not differ between the populations (no drought-by-population interaction: *F*_4, 940_ = 0.396, *p* = 0.811) (Table 2, Figure S2b). By day 42, shoots of drought treated plants were 15.9% smaller than control watered plants, but shoots of plants from the high elevation Big Horn Mountain population were still 19.0% larger than the Black Hills population. Shoot growth rates remained positive, even towards the end of the drought period (e.g., between days 43 and 51) for both the high elevation (0.61 ± 0.080 mm/day) and low elevation (0.44 + 0.079) populations.

### 2.4. Population Divergence

**Figure 2:**
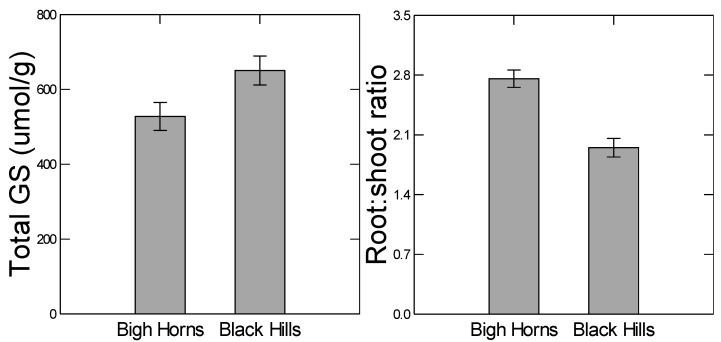
Genetic divergence between high and low elevation populations in basal dry mass root:shoot ratio and total glucosinolate concentration. Values are least squares means. Statistical analyses in Table 3 and Table 4. Error bars are ±1SE, total sample size *n* = 175.

As previously reported [11], high and low elevation populations of *B. stricta* showed genetic divergence with respect to total glucosinolate production and root:shoot ratio; neither population had high values of both kinds of traits (Figure 2). The high elevation population was higher in inherent dry mass root:shoot ratios across drought treatments (Population effect: *F*_1, 166_ = 27.522, *p* < 0.001). However, the 41.4% higher ratio under control watering of the high elevation population decreased to 25.8% under drought conditions (drought-by-population interaction: *F*_1, 166_ = 5.742, *p* = 0.018) (Table 3). The difference occurred because of a greater decline in the high elevation population. By contrast, the high elevation population had 20.8% lower inherent total glucosinolate levels (*F*_1, 162_ = 18.138, *p* < 0.001), a difference that was not affected by the environmental treatments of drought and herbivory (no treatment-by-population interaction: *F*_3, 162_ = 1.728, *p* = 0.163) (Table 4).

### 2.5. Principal Component Analysis of Drought Tolerance Traits

Several other trait measures in addition to growth, Betacyannin color score and root:shoot ratio were made to help assess evolutionary and ecological tolerance responses to drought. In addition, we measured glucosinolate production in several ways besides total glucosinolate content. Thus, we used multivariate principle component analysis to further assess the differences between populations in responses to drought and then herbivory.

Principal component analysis of 10 traits that may contribute to drought tolerance resulted in four significant PCs, each explaining at least 10% of the total variance (Table 5A). However, only for PC1 and PC4 were there effects of the environmental treatments or population line (Table 5B). PC1 was mainly positively correlated with carbon isotope ratio and negatively correlated with root:shoot ratio and LMA, while PC4 was mainly correlated with trichome density and stomata size (see component loadings, Table 5A). Although the significant effects on these PCs were attributable to the drought treatments and population line, but there was no interaction between these factors (Table 5B, Figure S3). An exception was for the separate analysis of the carbon isotope ratio.

**Figure 3:**
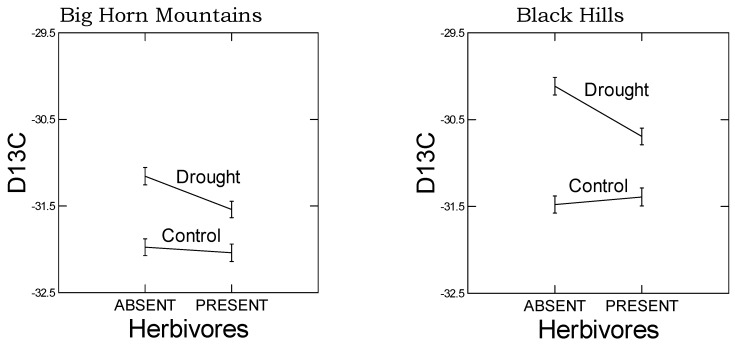
Effects of herbivory and drought on the carbon isotope ratio. See Table 5B for statistical analysis. The effect of herbivory was only significant in the drought treated plants (LSD multiple comparisons *post hoc* test: *p*’s < 0.05). Error bars are ±1 SE, total sample size *n* = 116.

In a separate analysis of the carbon isotope ratio (D13C, Table 5B), a main component of PC1, there was a significant effect of herbivory, but only in the drought treated plants (Figure 3). The effect of herbivory was to decrease D13C in the drought treated plants, an effect that was more dramatic in the Black Hills population (Table 5B, environmental treatments-by-population line interaction: *F*_9, 95_ = 3.114, *p* = 0.003).

### 2.6. Principal Component Analysis of Glucosinolate Measures

**Figure 4:**
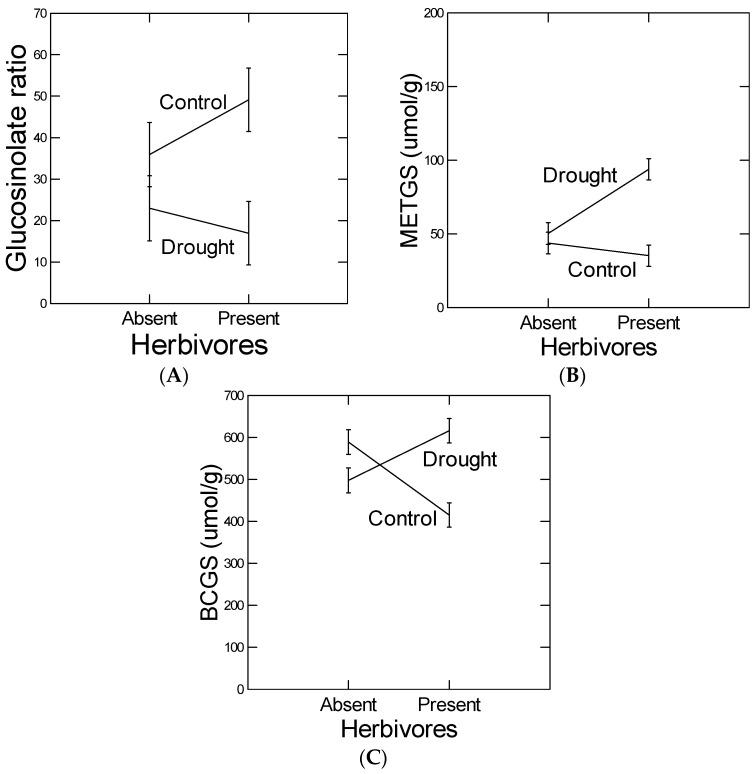
Effect of drought on the herbivore-induced responses of (**A**) ratio of branch chain (BCGS) to Methionine-derived straight-chain glucosinolates (METGS), (**B**) METGS, and (**C**) BCGS. Statistical analysis in Table 6. Error bars are ±1SE, total sample size *n* = 175.

Principal component analysis (PCA) of GS resulted in two significant PCs (Table 6A). The main components of PC1 included the GS ratios and the MET-GS 6-mthylsulfinylhexyl, whereas the main components of PC2 included the BCGS 2-hydroxyl-1-methylethyl and 1-methylethyl. Although there were significant differences among the population lines in all measures of GS production, the responses to herbivory and drought (Figure 4) did not depend on population line (no treatment-by-population line interaction, Table 6B).

### 2.7. Correlation between Carbon Isotope Ratio and Glucosinolate Production

There was a negative correlation between carbon isotope ratio and glucosinolate concentration that depended on environmental treatments and population (Table 7). In the Black Hills population, drought, but especially herbivory, was associated with the negative correlation. This was evident for BCGS (Figure S4A) or METGS (Figure S4B). In the high elevation Big Horn Mountain population, the correlation was negative across treatments, but the pattern was complicated by variation among the population lines. We did not have the statistical power to also include the lines in the statistical analysis. In contrast to the negative correlations involving GS concentrations, the correlation was positive for GS ratio (Figure S4C).

### 2.8. Gene Expression

**Figure 5:**
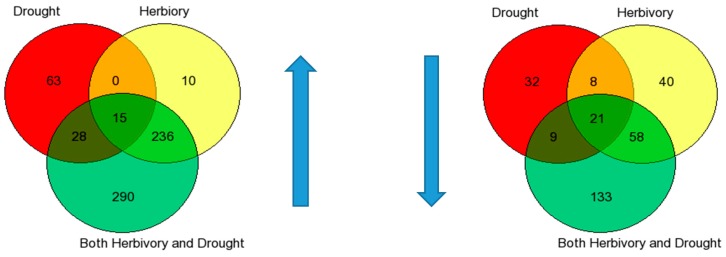
Venn diagrams for number of significantly up- and down-regulated genes (direction of blue arrows indicate up- or down-regulation). Red circle is Drought *vs.* control (no stress), Yellow circle = Herbivory *vs.* control (no stress), Green circle is double challenge Drought + Herbivory *vs.* control (no stress). Number of biological replicates, *n*, in comparisons: red circle, drought *vs.* control (*n* = 8); yellow circle, herbivory *vs.* control (*n* = 6), green circle, double challenge drought + herbivory *vs.* control (*n* = 8).

Drought and herbivory treatments were not additive for the number of genes differentially expressed. This was evident from the relatively high number of unique genes expressed in the double challenge drought/herbivore treatment combination (Figure 5). There were 290 (82.4%) unique genes up-regulated and 133 (79.2%) unique genes down-regulated in the double-challenge condition. Because of the high overlap between herbivory and the double challenge treatment, we assume that the response to herbivory alone was not blocked by drought. By contrast, because of the low overlap between drought and the double challenge combination, we assume that herbivory could have blocked some of the genes expressed in the drought alone treatment. To better understand the potential function of the unique genes expressed in the double challenge combination, the comparison was also made with single-challenge controls. Relatively more unique genes were expressed in the double-challenge treatment combination when the single-challenge control was drought (Figure S5), suggesting that the drought treatment could have enhanced the response to herbivory.

A functional analysis (gene ontology enrichment analysis) of the unique up-regulated genes in the double-challenge combination of drought and herbivory included 262 *Arabidopsis* analogs. Of these, 40.0% or 105 genes were involved in responses to abiotic and biotic stressors. But there was a greater proportion of defense (39.0%) and wound-related genes (28.5%) compared to water deprivation (18.1%). A similar functional analysis was conducted on 119 uniquely down-regulated *Arabidopsis* analogs. Of these, 25.2% or 30 genes were involved in biotic and abiotic (water deprivation) stress responses; none in defense and wounding, and 33.3% involved in water deprivation. Thus, the prolonged drought treatment up-regulated unique defense genes in response to herbivory, but the herbivory down-regulated unique water deprivation genes in drought-treated plants.

**Figure 6:**
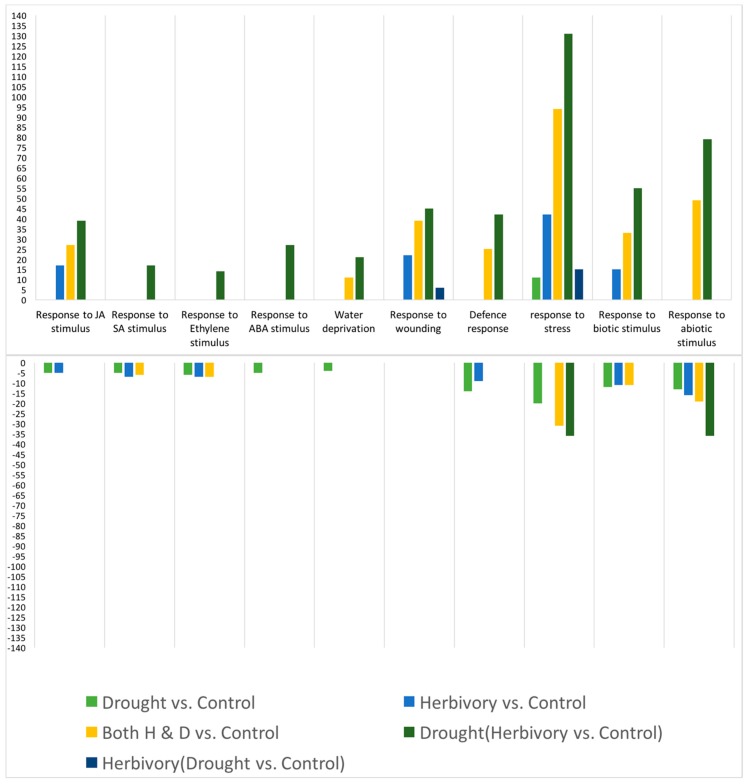
Number of significantly up- and down-regulated genes by defense and drought-tolerance functional categories. Herbivory (Drought *vs.* Control) means that both drought and control-watered treatments were fed upon by herbivores. Similarly, Drought (Herbivory *vs.* Control) means that plants in the presence and absence of herbivores were under drought stress. Number of biological replicates for the comparisons ranged between 6 and 10.

When the number of genes differentially regulated was examined by defense- and drought tolerance-related gene categories, some other notable patterns were evident (Figure 6). For example, although genes for JA signaling (e.g., LOX, JAZ—see heat map, Figure S6a for other examples) were up-regulated in response to *S. exigua* larvae feeding, defense response genes (e.g., CYPs, WRKYs—see Figure S6b for other examples) were down-regulated (Figure 6—light blue bar). However, under conditions of drought, herbivory up-regulated both JA signaling and defense related genes (Figure 6—darker green bar). Thus, drought apparently blocked the inhibitory effects of the herbivore to regulate the plant’s defense response. Of particular note is the dependence of the CYP79 herbivore-induced response on the presence of drought. The *B. stricta* homolog of CYP79 is involved in the GS-ratio response [19].

Signaling pathways that may mediate the apparent inhibitory effect of the herbivore and release of this inhibition by drought were identified in the hierarchical clustering analysis (Figure S6a). These candidate genes (transcription factors—TFs) were up-regulated in response to herbivory, but down-regulated when herbivores fed on drought stressed plants. Or, in the case of candidates involved in the ability of drought to block the inhibitory effects of the herbivore, activated only in the double challenge situation. These candidate TFs that were up-regulated by the herbivore, but down-regulated in the herbivore-drought combination, and their respective signaling pathway (in parentheses), included MYB95 (ETH), MYB13 (SA, ABA), AIB (ABA), and GRP-5 (ABA). However, all of the pathways had some TFs that were only up-regulated in the double challenge, such as MYC2 (JA), that could be involved in the blocking of the herbivore inhibition of the defense response. TFs such as MYC2 have been previously implicated in the crosstalk between responses to biotic and abiotic stressors [14]. MAPK signaling was also implicated in a similar analysis using gProfiler. MAPK signaling may be involved in the regulation of coordinated signaling responses under abiotic and biotic stress [20].

**Figure 7:**
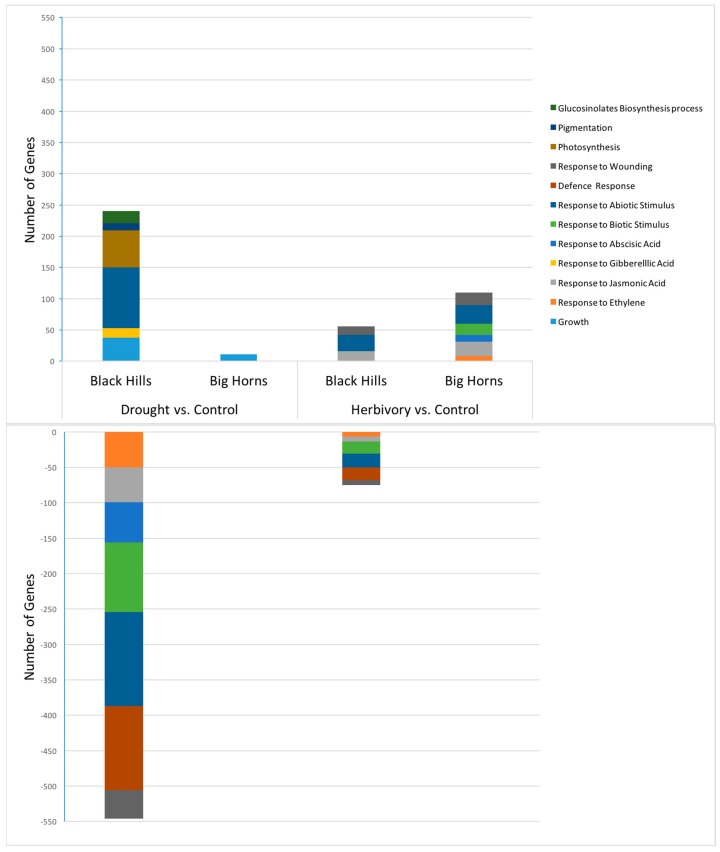
Number of differentially expressed genes of relevant biological processes between populations. The number of biological replicates for the Black Hills population comparisons was *n* = 4 and for the Big Horn population, *n* = 5.

Finally, a comparison between high and low elevation populations for the number of relevant differentially expressed genes indicated that the low elevation population was more responsive to drought, as predicted, while the high elevation population was more responsive to herbivory, as also predicted (Figure 7). While this was clear for up-regulated genes, it was, however, less clear for down-regulated genes. 

## 3. Discussion

Many factors and processes, alone or in combination, may contribute to species range limits development by preventing adaptation to stressful environments. Some major factors include lack of genetic variation in range margin populations, barriers to dispersal, swamping gene flow from elsewhere within the range and various kinds of tradeoffs [2]. Of these, relatively little is known about possible molecular, physiological or developmental tradeoffs [5].

Here, we tested predictions from a hypothesis explaining the existence of an apparent genetic tradeoff between defense allocation and abiotic stress tolerance. This tradeoff may contribute to low elevation range limit development. The hypothesis states that antagonistic plastic response pathways may inhibit their simultaneous co-option for more stable expression that is needed for range expansion. If a plastic response pathway in a signaling network is co-opted in evolution for more stable expression, then one might predict that other pathways in the network would also be affected [13]. To test this prediction, we compared two genetically diverged populations for their plastic responses to drought and herbivory. For example, the high elevation population from the Big Horn Mountains has diverged with respect to higher constitutive levels of some abiotic stress tolerant traits, such as root:shoot ratio (Figure 2). Since the root:shoot ratio is in part regulated by ABA signaling [21], we tested the prediction that ABA-regulated traits, such as stomatal aperture, would be less plastic in the high elevation population. We also expected there to be less gene expression in response to drought in the high elevation population. Similarly, we expected the low elevation population to be less plastic in defensive traits. In general, we predicted that relatively high constitutive levels of the functional traits would coincide with lower plasticity.

Although our central hypothesis involved evolutionary constraints caused by potentially antagonistic signaling pathways, we did not make direct measurement of the pathway hormones, making the link between the signaling and the ecological and evolutionary responses speculative. However the predictions that we tested did not necessitate direct measurement of the hormones that trigger the candidate pathways. Instead, we relied on existing literature that makes a link between the candidate signaling pathways, gene expression and the functional traits. The candidate signaling pathways that were invoked for the evolutionary tradeoff between chemical defense allocation and stress tolerance in *B. stricta* were the jasmonic acid/ethylene (JA/ET) and the abscisic acid (ABA) pathways, respectively. These pathways are generally antagonistic to one another in *Arabidopsis*, probably because a stress response under dry conditions takes precedence over defense that may function primarily against pathogens under moist conditions [14,15,16,20] for reviews). It is also known that JA/ET signaling regulates aliphatic glucosinolate (GS) toxin-induced defense responses in *Arabidopsis* [22,23], induced resistance against generalist insect herbivores in another closely related species *Boechera divaricarpa* [24], and that GS are defensive against generalist insect herbivores as has also been found to be the case for *B. stricta* (e.g., [7,19]). We therefore assumed that JA/ET signaling regulates aliphatic GS defense responses to generalist insect herbivores in *B. stricta*. Likewise, ABA is a general stress response hormone, produced most notably in response to abiotic stressors, especially to drought or salinity, but also to other factors such as nutrient deficiency ([21,25] for reviews, [26]). For example, in response to limiting supplies of water or soil nitrate levels and subsequent increases in ABA concentration or sensitivity, stomata close, leaves grow more slowly, and root growth is maintained and characterized by lateral root proliferation. Whether the genetic divergence between high and low elevation populations in levels of aliphatic GS or the various other functional traits associated with abiotic stress tolerance that we measured represents genetic divergence in the joint JA/ET regulatory pathway or ABA signaling was tested here indirectly without direct measurement of the hormones.

As predicted, in the high elevation population, we observed a reduced plastic stomatal aperture response to drought as measured by the carbon isotope ratio (Figure 3). That is, for the carbon isotope ratio, the difference between drought and control treatments was smaller in the Big Horn Mountain population compared to the Black Hills population. However, less regulation on stomatal control is typical of alpine plants where water deficiency is usually not a problem [27]. The drought-induced change in the root:shoot ratio was actually greater in the high elevation population, which was not predicted. However, the change was a decrease in the root:shoot ratio, which may not be mediated by ABA signaling. Under natural circumstances, root:shoot ratios increase in response to drought, but gradual water deprivation in small pots when watered from above is not conducive to increases in root:shoot ratio. Yet there was also no population-by-drought treatment interaction in the ANOVA for any of the four significant PCs constructed from the 10 putative abiotic stress tolerance response variables (Table 5B). This interaction would be an indication of any differences in plasticity between the populations. It was not until we analyzed the gene expression data that there was relatively clear evidence of differential plasticity in response to drought between the populations. For the number of relevant up- and down-regulated genes, the high elevation Big Horn population was indeed less plastic to drought.

In a similar study, transcript profiles resulting from cDNA-AFLPs were compared between high and low elevation populations of *Boechera holboellii* in a dry-down growth chamber experiment [28]. The focus was on identifying candidate genes involved in local adaptation by noting population-specific expression patterns. Although the dry-down treatments resulted in differential expression patterns, only a couple of *Arabidopsis* homologs were identified as candidates. Probably because of the method used for transcript profiling, there were too few gene expression differences between populations that were reported to evaluate predictions of our hypothesis.

Similarly, because the low elevation Black Hills population was higher in constitutive levels of total glucosinolate content (Figure 2), we predicted lower plastic responses to herbivory from that population. However, we did not detect any population differences in herbivore-induced glucosinolate measures (no indication of any herbivory-by-population interaction in the ANOVAs, Table 4 and Table 6). Many other studies have checked for negative genetic correlations between constitutive and herbivore-induced defense or resistance levels. This is because popular theory on the evolution of herbivore-induced defenses assumes resource allocation costs of constitutive defenses and cost-savings of inducible defenses ([29] and references therein). The results have been mixed, probably because several factors may influence constitutive and induced defense levels [30]. In response to herbivory, the low elevation Black Hills population was clearly less plastic in terms of the number of relevant up-regulated genes expressed, but this was not the case for the number of down-regulated genes.

Our predictions were based on the existence of antagonistic defense and abiotic stress tolerance plastic responses in *B. stricta*, as has been well documented in *Arabidopsis* [20]. Specifically, we checked whether a glucosinolate defense response to the generalist insect herbivore *S. exigua* was negatively affected under conditions of water deficiency. The assumption that we made was that under drought conditions, ABA signaling would interfere with JA/ET signaling to attenuate herbivore-induced defense responses [14]. We found that an herbivore-induced GS ratio (BCGS/METGS) response was attenuated under conditions of drought. The BCGS/METGS ratio has been shown to cause resistance to generalist insect herbivores in *B. stricta* [19,31] and therefore may be the most relevant GS response variable. That is, in these other studies, this GS ratio in *B. stricta* has been fine mapped, and candidate genes have been transformed and near isogenic lines created and tested with positive results. Interestingly, feeding by *S. exigua* caused GS concentrations to decrease, which was reversed under drought (Figure 4; Note, BCGS and total GS concentrations are highly correlated r = 0.98 because of the relative abundance of BCGS). Because increased GS concentrations render plants more susceptible to specialists like flea beetles (e.g., [32]), we suggest that the plants responded to feeding by increasing resistance to both generalists (by increasing GS ratio) and specialists (by decreasing total GS concentrations). If so, the drought treatments were antagonistic to herbivore-induced defense responses. To our knowledge, it is not known why the GS ratio is effective independent of total GS concentration.

In apparent contrast to the GS results, the gene expression results suggested that the *S. exigua* larvae may down-regulate the *B. stricta* induced defense response, but that this inhibition by the herbivore was blocked by the drought treatments. Of course, to verify this suggestion, direct hormone, proteome, metabolome and other experimental molecular and genetic analysis would help. Nonetheless, although an herbivore-induced JA response was evident, a defense response was notably absent (Figure 6). Patterns of gene expression further suggested that the putative herbivore inhibition may be mediated by other pathways such as ET, SA and ABA. That herbivores may inhibit defense responses downstream of JA signaling via an ethylene burst was reported previously for feeding by the specialist herbivore *Manduca sexta* on wild tobacco *Nicotiana attenuata* [33]. Here, we show that apparent signaling-mediated manipulation of plant defense responses by herbivores may be dependent on abiotic conditions. The mechanism of drought to block the herbivore inhibition of defense apparently involved further signaling crosstalk. However, in apparent agreement with the GS results, was the dependence of the CYP79 herbivore-induced response on the presence of drought. The *B. stricta* homolog of CYP79 is involved in the GS-ratio response [19].

A negative correlation was also observed between smaller stomatal apertures, as indicated by less negative carbon isotope ratios, and GS levels (Figure S4). Interestingly, this pattern held for both BCGS and METGS, but the correlation was positive for GS ratio. In addition, these correlations were dependent on drought and herbivore treatments, the particulars of which differed between populations (Table 7) and probably lie within populations. For example, in the low elevation Black Hills population, the correlations were dependent on the presence of drought and especially herbivory. What caused these correlations? These correlations may be dependent on the area of leaf tissue consumed, which we did not record, but which presumably would have influenced water loss and stomatal behavior. Alternatively, these correlations may reflect interactions between hormone-mediated response pathways.

## 4. Experimental Section

### 4.1. Study System

*Boechera stricta* (Brassicaceae) is a monophyletic, predominantly self-fertilizing, diploid, genetically diverse perennial and close relative of *Arabidopsis thaliana* that ranges across western North America at higher altitudes [34,35,36,37]. Unlike *Arabidopsis* in North America, *B. stricta* and many other species of *Boechera* are native, occur in natural habitats, and because of longer life cycles, face, and presumably adapt to, more ecological stressors [38,39,40]. Here we focused on plastic responses of genetically diverged high and low elevation populations of *B. stricta* in a double challenge experiment; herbivore induced defense responses under prolonged laboratory experimental drought.

The high elevation population was located at 44°18′22″N, 107°18′33″W, elevation 2780 m in the Big Horn Mountains, Wyoming and the low elevation population 44°24′50″N, 103°56′18″W, elevation 1365 m, the Black Hills, South Dakota. These are geographically isolated and genetically divergent populations [9,35]. The populations are located at different ends of the altitudinal range of *B. strict*, typically 1700 to 3000 m [36], and thus the sites differ by several environmental factors.

### 4.2. Growth Chamber Experiment

A double-challenge growth chamber experiment was conducted to determine the effects of drought stress on plant defense responses to herbivores. In nature, plants experience slow increases in drought stress, but encounter shorter bouts with herbivores. Therefore, we slowly increased plant exposure to more severe water deficiency (drought) and then challenged the plants with a relatively short bout of attack by herbivores. Of particular interest was the comparison of high and low elevation populations of *B. stricta*. As detailed below, the 4-week long drought treatment consisted of progressively reduced amounts and frequencies of watering for a gradual soil dry-down that slowly brought the plants close to the wilting point. During the gradual drought treatment process, plants were frequently monitored non-destructively for stress by examining decreased growth rates and color changes indicative of antioxidant production. These assessments of plant status determined the amount and frequency of watering treatments, which was recorded by weighing planting flats. After a brief 3-day recovery period where plants were brought back to a slightly more mild level of stress, early instar larvae of the generalist herbivore *Spodoptera exigua* were allowed to feed on stressed and control plants over a 2-day period to induce a glucosinolate defense response. At the end of the experiment, plant tissue was analyzed for several additional drought-tolerance related traits: carbon isotope ratio (water use efficiency—WUE), leaf trichome and stomata size and density, root:shoot mass ratio, leaf mass area (LMA) and the number of leaves per rosette. Glucosinolate levels were measured to assess defense response. On a subset of plants, whole-genome gene expression was also examined to identify signaling pathways involved.

#### 4.2.1. Experimental Design

The design of the growth chamber experiment was split-plot [41]; drought and herbivory treatments varied among planting flats, while population varied within flats. There were 256 plants total in the experiment distributed among eight flats, each containing 32 pots. We used two uniparental lines per population, and we randomized eight replicates of each line within each flat (2 pops/flat × 2 lines/pop × 8 plants/line = 32 plants/flat). Seeds were planted in 0.2-L pots filled with a soil mix of 2/3 *Premier ProMix BX* and 1/3 sand. Pots also contained 45.78 mg of 7:40:6 NPK MagAmp time release fertilizer. Plants were grown in a BioChambers growth room with a 16/8 h D/N photoperiod and 23/21 °C D/N temperatures. Light intensity was 360 µmoles/m^2^/s from a combination of 1220 mm T5HO fluorescent and halogen lamps.

Watering and herbivory varied among flats for practical reasons. Drought treated flats were watered less and less often (see below), which was monitored by weighing whole flats with a postal scale rather than weighing pots individually. Similarly, although individual plants were caged (see below), to reduce effects of any caterpillars moving between plants within a flat, flats either had no caterpillars or had caterpillars on all plants. However, because plants were in separate pots with their own soil and water, and because caterpillars were caged on individual plants, individual plants were otherwise treated independently of one another.

#### 4.2.2. Drought Treatments

Watering treatments began on day 26 after planting, and herbivore induction treatments on day 54. When watering treatments began, all flats were also watered with 0.7 g/500 mL 20:20:20 NPK + micronutrient Peter’s fertilizer. For the control and drought watering treatments, the experiment was divided into two groups of four flats each. There was no difference in average rosette diameters among the two groups of flats (*F*_1, 6_ = 0.058, *p* = 0.817) when watering treatments began.

During the period prior to herbivore feeding, drought treated plants were gradually stressed such that growth rates decreased relative to controls, but remained positive. While control flats were watered to 9 kg every four days, the drought treated flats were watered less and less often in a progressively decreasing manner. The amount of water was monitored by flat weights (see Results). The same amount of water was distributed among pots within flats by moving with the watering can across flats at a constant rate.

#### 4.2.3. Plant Growth Rates

In the process of decreased turgor and subsequent ABA synthesis caused by water deficiencies, growth decreases before photosynthesis and subsequent wilting [42]; therefore, rosette size was used to help monitor effects of the watering treatments and to determine the amount and frequency of watering treatments. Thus, watering treatments were administered such that drought treated plants grew slower than did controls. Rosette size was measured using digital calipers beginning as soon as true leaves appeared and continued every 9 to 10 days during the experiment.

#### 4.2.4. Leaf Betacyannin Production

Additionally, leaf color change as an indicator of antioxidant production, and any signs of wilting (drooping rosette leaves) were also used to monitor drought treatments. Under drought stress, *B. stricta* leaves turn a red violet color because of Betacyannin production. The color change is mainly on the lower epidermis of the leaves. Betacyannins are pigments that are produced in response to abiotic stress such as drought and function as ROS scavengers [43]. The Betacyannin response of an individual plant was recorded visually on a subjective scale (0, 1, 2, 3) corresponding to the extent of the red violet coloration of the leaves.

#### 4.2.5. Herbivore Induction Treatments

Larvae of the generalist insect herbivore *Spedoptera exigua* (Beet armyworm) were used to induce a chemical defense response in *B. stricta* plants. In particular, we assumed that aliphatic glucosinolate levels would change in response to *S. exigua* feeding as can occur in *Arabidopsis* [44]. Eggs from Benzon Research were hatched onto cabbage in Styrofoam cups and allowed to feed before being placed on plants. Four early instar caterpillars were place on each plant in two of the flats in each watering treatment. Individual plants were caged in clear plastic cylinders (*i.e.*, clear plastic cups with the bottom cut out and wedged into the soil around the plants) covered with netting. Plants in herbivore-control plants were also caged to control for any cage effects. After two days of feeding, the experiment was terminated for plant tissue harvest.

#### 4.2.6. Water Use Efficiency

Water use efficiency is a ratio of CO_2_ uptake to water loss, but the carbon isotope ratio (δ^13^C) is also used to estimate WUE in C_3_ plants ([45] and references therein, [46]). This is because the ^13^C/^12^C ratio can be modeled as a function of the ratio of intercellular to atmospheric partial pressure of CO_2_ (C_i_/C_a_), which is also supported empirically, and C_i_/C_a_ is empirically correlated with WUE in C3 plants. Values of δ^13^C are usually negative, and less negative values indicate greater WUE. For carbon isotope discrimination, whole basal rosette shoots were freeze-dried, ground to <0.5 mm and analyzed on a Thermo Delta V isotope ratio mass spectrometer (IRMS) interfaced to a NC2500 elemental analyzer at the Cornell Isotope Laboratory (COIL). Values were expressed as per mL (‰) ^13^C values.

#### 4.2.7. Leaf Trichomes and Stomata

Trichome and stomata size and densities are known to affect plant water relations in montane plants [27]. Cell size and densities were recorded from freeze-dried leaves using a compound light microscope and fingernail polish leaf peels of the lower epidermis. We used fully expanded leaves near the center of rosettes. One leaf from each plant was used, but two measures, one on either side of the leaf midrib, were averaged for each leaf. Stomata cell size was measured from a length measurement at 400× magnification, and stellate trichome cell size was calculated from length and width measures at 100×. Cell lengths and widths were measured using a calibrated ocular micrometer. The number of stomata cells were counted in the entire field of view at 400×, which was an area of 0.196 mm^2^, while trichome counts were conducted at 100×, and area of 0.314 mm^2^. The areas allowed us to convert the counts to densities.

#### 4.2.8. Root:Shoot Ratio and Number of Leaves

At the end of the experiment, fresh and freeze-dried weights were obtained for whole shoots (basal rosettes) and roots. Roots were floated in water and rinsed to remove sand and soil materials before weighing and freeze drying. Shoots and roots were flash frozen in liquid nitrogen before freeze-drying. The number of leaves per rosette were also counted from freeze-dried shoots.

#### 4.2.9. Glucosinolates

Glucosinolates were extracted in methanol, isolated on Sephadex ion-exchange columns, and measured on a HPLC [47,48] as summarized elsewhere [10]. Briefly, weighed, freeze-dried basal rosette leaves were extracted in 1.2 mL methanol, separated on a 0.6-mL DEAE A-25 Sephadex column, and eluted after 12 h incubation with sulfatase (Sigma-Aldrich, St. Louis, MO, USA). A Lichroshpere (RP-C18, endcapped) 250 × 4-mm analytical column was used on the HPLC, and chromatograms generated at 229 nm were analyzed.

#### 4.2.10. Statistical Analysis

We used SYSTAT version 13.0 for all statistical analyses of defense and drought tolerance phenotypes. To analyze the split-plot statistical model, we used mixed-model (*i.e.*, the presence of fixed and random effects) ANCOVA with Type III sums of squares. For example, we used the model

Response = C + Drought + Flat (Drought) + Pop + (Drought × pop) + size

in the analyses of the effects of drought treatments and population. Response represents the response variable (e.g., carbon isotope ratio), C = constant, Flat (Drought) represents unmeasured random variation among flats not accounted for by the whole-flat treatment factors (e.g., drought), Pop is for population (*i.e.*, high or low elevation populations) and size represents seedling size, which was included to control for any initial developmental differences. In this case, drought and population were fixed effects and seedling size was a random covariate effect. We also conducted repeated measures ANCOVA when using rosette diameters and as the response variables. We computed *F*-ratios from appropriate mean-square errors for this split-plot design (Zar 1996, [49]). Preliminary inspection of the data for the interaction between population or population line and any unmeasured whole-flat factors (population-by-Flat (Drought)) determined that this interaction was not important; therefore this interaction was not included in the analyses (Montgomery 1997, [50]). Eliminating this interaction simplified the analyses and did not affect the main results. For example, in the above model, the *F*-ratio for Drought was calculated using the Flat (Drought) mean square error in the denominator, otherwise for the other factors, F-ratios were calculated over the residual mean square error.

Principal components were constructed to reduce the dimensionality of drought-tolerance-related variables, of which there were *n* = 10, and of glucosinolate measures, of which there were *n* = 7. Essentially, SYSTAT uses the rule of thumb that PCs are significant if they explain at least 1/*n* of the variance [51]. Significant PCs were used as response variables in the split-plot ANCOVA modes (see previous paragraph).

#### 4.2.11. Gene Expression

Shoots of 13 plants representing all four treatment combinations (presence and absence of drought and herbivory) and the two populations were used for RNAseq analysis. There were at least 2 to 3 replicates per population in each herbivory treatment (presence, absence) under drought conditions, but there were just 1 to 2 replicates per population in the herbivory treatments under control watering conditions. This unbalanced design occurred because of funding limitations. The replicates within populations were from the same line; line 63 from the Big Horn Mountains and line 48 from the Black Hills.

QIAGEN RNeasy Mini Kit was used for RNA extraction. In brief, plants samples were crushed/powdered in liquid nitrogen and 33 mg of the powder was extracted RLT buffer. After precipitation, RNA was purified using DNase digestion and washes. RNA quality was checked using a Qubit Fluorometer and a denaturing gel.

Extracted RNA samples were sent to DHM (David H. Murdock) Research Institute for Illumina sequencing. RNA libraries were constructed for each sample and each uniquely tagged with a molecular barcode or “index”. Those libraries were quantified using Real Time PCR. Two pools were generated from these libraries and sequenced via a 100 base pairs single read sequencing run on the Illumina HIseq2500 platform.

Data analyses were performed by utilizing tools in Discovery Environment, iplant collaborative (https://de.iplantcollaborative.org/de/). Trimmed quality reads obtained from BHM Research Institute were mapped to *Boechera stricta* reference genome utilizing Tophat2 tool. Max intron length used in Tophat2 was 5000, while the default setting was used for other parameters. *Boechera stricta* gene annotation file (Gff3 format) was used; Tophat2 aligns the sequence to the transcriptome first, then only unmapped files are aligned to the reference genome. Cufflinks2 was used to build the transcripts by using each Tophat2 mapping file, which was further used in Cuffmerge2 to merge the newly identified transcripts with already predicted transcripts from *Boechera stricta* genome. The merged file with reference ids (gtf format) was used as annotation in Cuffdiff2 along with individual mapping files (bam files by Tophat2) from every sample to examine the differential expression of genes due to comparison of treatment with control. From the output of differentially expressed genes provided by Cuffdiff2, only the genes with Log2 fold change ≤−2 or ≥2 and false discover rate of 0.05 was considered for further analysis.

AGRIGO Singular Enrichment Analysis tool was used for GO enrichment analysis of differentially expressed genes in individual treatments. For examining the number of genes involved in various biological processes related to drought and herbivory in different treatments, we used gprofiler (http://biit.cs.ut.ee/gprofiler/gcocoa.cgi). Venn diagrams were made by utilizing “Genevenn tool” (http://genevenn.sourceforge.net) for examining overlapping of genes in different treatments. Hierarchical clustering of genes related to drought and herbivory was performed using Cluster 3.0 software by using complete linkage function. Output files produced by Cluster 3.0 were used in Java TreeView to visualize Hierarchical clustering maps.

RNAseq data from this study have been submitted to the NCBI Gene Expression Omnibus (GEO; http://www.ncbi.nlm.nih.gov/geo/) under accession number GSE78101’.

## 5. Conclusions

Although it is now relatively well-known that plant responses to simultaneous challenges of biotic and abiotic stressors are not additive and involve signaling crosstalk [20], much less is known about the evolutionary implications of these interactions. Here, we tested a hypothesis for range limit development involving these interactions. The hypothesis states that antagonistic crosstalk between signaling pathways may be an evolutionary constraint, preventing adaptation to stressful environments across the range boundary. The constraint occurs if the crosstalk inhibits the simultaneous co-option of the antagonistic response pathways. For example, evolution of transcription factors involved in the crosstalk may have negative pleiotropic effects. We tested the hypothesis by comparing populations that have diverged with respect to defense and drought tolerance traits as one would predict if natural selection acted on antagonistic signaling pathways; neither population was high in both kinds of traits—defense and stress tolerance. The populations that had diverged with respect to traits regulated by the abiotic or biotic stress response pathways were in some cases less inducible, as we predicted based on the central hypothesis; however the results were ambiguous, depending on the level of analysis. That is, while some support was found at the level of gene expression, relatively little support was found in the analysis of trait phenotypes. We therefore suggest that genetic assimilation ([52]) of signaling pathways and the evolutionary consequences of crosstalk may be better studied at the molecular level. Recent modeling efforts predict that range shift response to climate change may be more pessimistic when variation among populations in phenotypic plasticity is accounted for [53]. While some studies indicate that complex traits controlled by signaling networks may be inherently evolutionarily constrained [17], others do not [54]. Thus, more work is needed to simultaneously connect molecular, evolutionary and ecological contexts for plant responses to biotic and abiotic stressors.

## Figures and Tables

**Table 1:** ANOVA for violet red “Betacyannin” leaf color score. Census was taken on 30 December and 14 January and then the analysis was conducted on the cumulative score. *r*^2^ = 26.8%.

Source	df	Mean Squares	*F*-Ratio	*p*-Value
Population	1	0.064	0.069	0.793
Drought	1, 6	57.012	15.187	0.000
Drought × pop	1	0.125	0.135	0.713
Flat (Drought)	6	3.754	4.059	0.001
Error	235	0.925		

**Table 2:** Repeated measures analysis of shoot size recorded five times during the experiment.

**Between Subjects**					
**Source**	**df**		**Mean Squares**	***F*-Ratio**	***p*-Value**
Population	1		1453.863	31.175	0.000
Drought	1, 6		3394.266	7.079	0.037
Drought × pop	1		1.850	0.040	0.842
Flat (Drought)	6		479.496	10.282	0.000
Error	235		46.636		
**Within Subjects**					
**Source**	**df**	**Mean Squares**		***F*-Ratio**	***p*-Value**
Day	4	20,292.390		1401.337	0.000
Day × Population	4	100.583		6.946	0.000
Day × Drought	4, 24	788.871		6.281	0.001
Day × Drought × Pop	4	5.738		0.396	0.811
Day × Flat (Drought)	24	113.572		7.843	0.000
Error	940	14.481			

**Table 3:** ANOVA of dry root:shoot mass ratio. *r*^2^ = 49.2%.

Source	df	Mean Squares	*F*-Ratio	*p*-Value
Population	1	45.266	37.725	0.001
Drought	1, 6	13.293	27.522	0.000
Drought × pop	1	2.773	5.742	0.018
Flat (Drought)	6	1.200	2.484	0.025
Seedling size *	1	4.950	10.249	0.002
Error	166	0.483		

* width of seedling across cotyledons.

**Table 4:** ANOVA of total glucosinolate concentration.

Source	df	Mean Square Error	*F*-Ratio	*p*-Value
Drought & Herbivory	3	314,545.772	7.271	0.000
Population	1	784,711.045	18.138	0.000
(D & H) × Pop	3	74,748.963	1.728	0.163
Flat (D & H)	4	131,583.387	3.041	0.019
Seedling size	1	141,881.984	3.280	0.072
Dry leaf wt	1	1,312,877.577	30.346	0.000
Error	162	43,263.071		

**Table:** (A)

Traits	PC1	PC2	PC3	PC4
Carbon isotope ratio	**0.721**	−0.428	−0.023	0.164
Betacyannin color score	**0.532**	0.106	**−0.490**	−0.051
Root:shoot ratio	**−0.694**	0.115	−0.228	−0.102
LMA	**−0.668**	−0.051	0.083	0.375
Trichome size	0.281	0.286	**0.539**	0.367
Trichome density	0.145	**0.513**	0.305	**0.508**
Stomata density	0.137	**0.577**	0.260	−0.439
Number of rosette leaves	**−0.476**	**0.483**	−0.335	0.101
Stomata length	0.220	0.172	**−0.689**	**0.456**
Growth rate	−0.395	**−0.649**	0.165	0.204
Variance explained	22.8%	15.7%	13.6%	10.2%

**Table:** (B)

Source	df	PC1	PC2	PC3	PC4	D13C
Drought & Herbivory	3, 4	17.823 **	0.333	0.009	0.847	9.284 *
Pop Line	3	17.276 ***	0.825	1.263	13.177 ***	30.023 ***
(D & H) × Pop Line	9	0.858	1.266	0.824	0.892	3.114 **
Flat (D & H)	4	4.641 **	5.258 ***	2.308	3.225 *	7.421 ***
Seedling size	1	2.049	8.170 **	6.926 **	1.101	5.353 *
Error	96					
*r*^2^		80%	34.9%	23.7%	44.1%	81.5%

* *p* ≤ 0.05; ** *p* ≤ 0.01; *** *p* ≤ 0.001.

**Table:** (A)

GS Measure	GS-PC1	GS-PC2
Total GS	0.653	0.655
2-hydroxyl-1-methylethyl GS (GS1)	0.502	**0.811**
1-methylethyl GS (GS2)	0.528	**0.748**
6-mthylsulfinylhexyl GS (GS3)	**0.996**	0.040
BCGS/METGS (GS1 + GS2)/GS3	**−0.861**	0.489
GS1/GS3	−0.861	0.477
GS2/GS3	−0.750	0.582
Variance explained	57.0%	35.0%

**Table:** (B)

Source	df	GS-PC1	GS-PC2	GS-Ratio	BCGS	METGS
Drought & Herbivory	3	6.606 ***	1.606	6.788 ***	6.309 ***	5.789 ***
Pop line	3	5.662 ***	45.930 ***	17.321 ***	14.882 ***	4.993 **
(D & H) × Pop line	9	0.437	1.410	0.459	1.075	0.409
Flat (D & H)	4	2.081	0.744	1.792	2.492 *	1.810
Dry leaf wt	1	13.178 ***	127.507 ***	1.086	33.259 ***	21.848 **
Seedling size	1	0.257	8.338 **	1.571	4.097 *	0.332
Error	154					
*r^2^*		33.6	68.9%	37.2%	50.8%	35.2%

* *p* ≤ 0.05; ** *p* ≤ 0.01; *** *p* ≤ 0.001.

**Table 7:** *F*-ratios from ANCOVAs on carbon isotope ratios. The analysis was conducted separately for different combinations of glucosinolates (BCGS—branch chain GS, METGS—methionine-derived GS, GSRATIO—ratio of BCGS to METGS) and population (BIHM—Big Horn Mountains, BLHI—Black Hills).

	df	BCGS		METGS		GSRATIO	
		BIHM	BLHI	BIHM	BLHI	BIHM	BLHI
GS	1	10.754 **	33.509 ***	32.322 ***	30.752 ***	26.655 ***	25.120 ***
Drought	1, 1	144,624 **	9.666	16.385	57.407	1.625	9.805
Herbivore	1, 1	3.502	0.480	2.198	10.304	1.879	31.006
Drought × GS	1	0.000	4.564 *	1.520	1.564	7.880 **	9.915 **
Herbivore × GS	1	0.130	7.761 **	0.091	0.356	1.124	0.311
Drought × Herbivore	1, 1	0.077	3.746	0.105	1.713	0.697	15.705
Drought × Herbivore × GS	1	1.967	0.375	0.458	9.766 ***	4.227 *	0.525
Error	82						
*r^2^*		34.3%	75.1%	50.7%	75.9%	45.6%	71.5%

* *p* ≤ 0.05; ** *p* ≤ 0.01; *** *p* ≤ 0.001.

## Supplementary Materials

Supplementary Materials can be found at www.mdpi.com/2223-7747/5/1/13/s1.
