# Supplementary material for: Effect of Drought on Herbivore-Induced Plant Gene Expression: Population Comparison for Range Limit Inferences
Source: Plants (Basel). 2016 Mar 11;5(1):13. doi: 10.3390/plants5010013 (PMC4844423; doi:10.3390/plants5010013)
Supplement: Supplementary file 1 [file plants-05-00013-s001.pdf]

Supplementary Materials: Effect of Drought on Herbivore-Induced Plant Gene Expression: Population Comparison for Range Limit Inferences

Gunbharpur Singh Gill, Riston Haugen, Steven L. Matzner, Abdelali Barakat and David H. Siemens

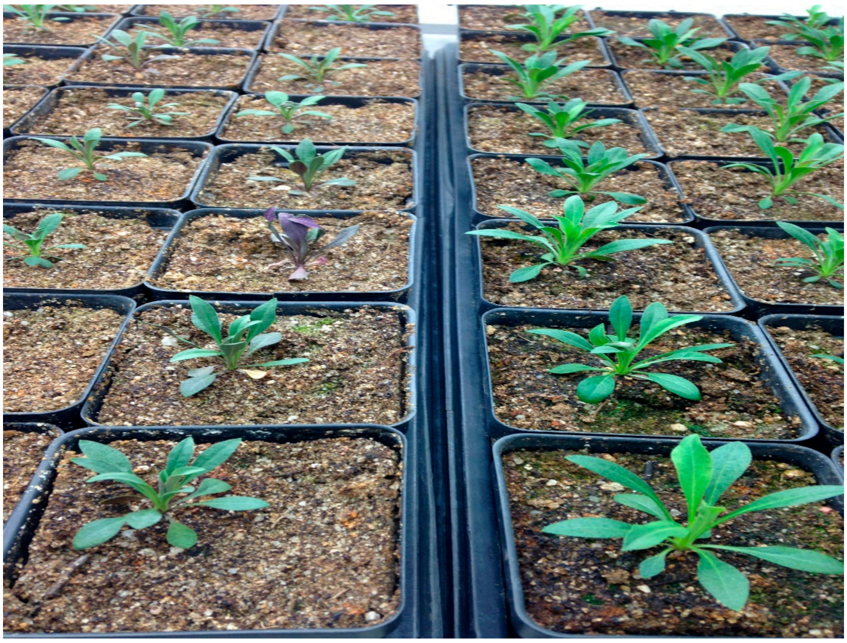

Figure S1. Examples of drought a drought treated flat (**left**) and control treated flat (**right**) on day 51 post-planting, 24 days after the drought treatments began. At this time flat weights of drought treated plants fell just below 6.5 kg (see Figure 1b), and two plants total in the drought treatment group had curled rosette leaves indicating early signs of wilting. One of the affected plants with curled leaves is indicated in the left of the photo by the white arrow. This indicated that plants in the drought treated group were experiencing stress.

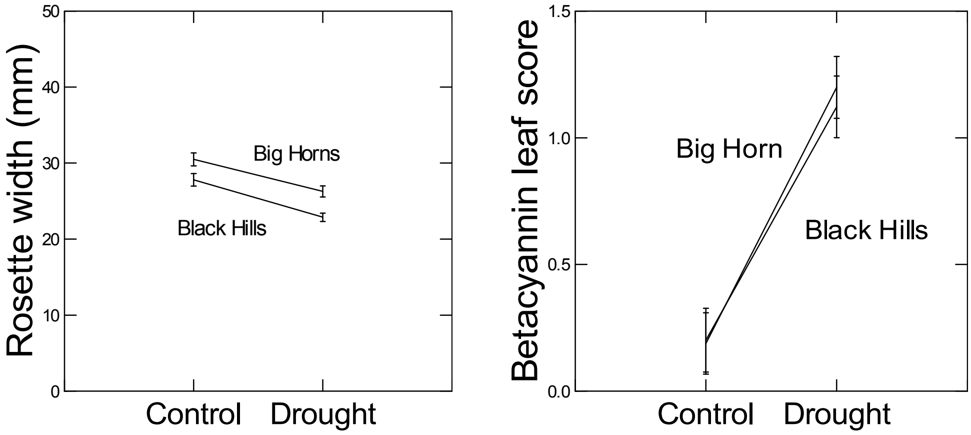

(**A**) (**B**)

Figure S2. Effects of drought treatment and population on (**A**) Betacyannin leaf color score; and (**B**) plant shoot size. Values are least squares means. See Tables 1 and 2 for statistical analysis.

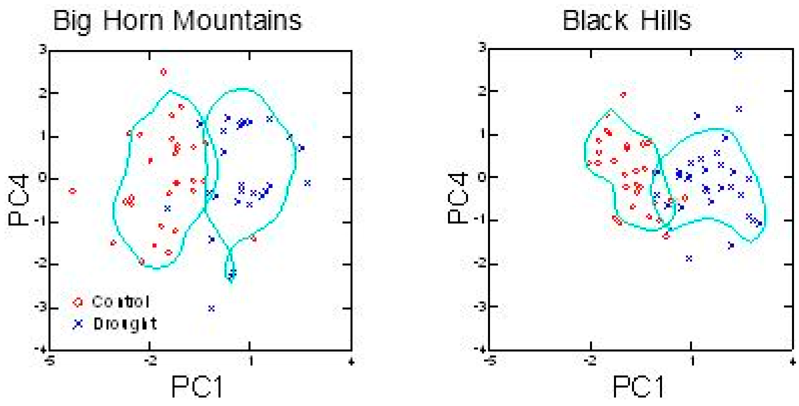

Figure S3. Drought stress tolerance PC bi-plots for the high and low elevation populations. See Table 5 for component loadings and statistical analyses. There was no effect of herbivory on PC1 (Fisher's Least-Significant-Difference Test: $p > 0.05$) and therefore are not shown; only the drought treatments were significant (LSD p's < 0.05).

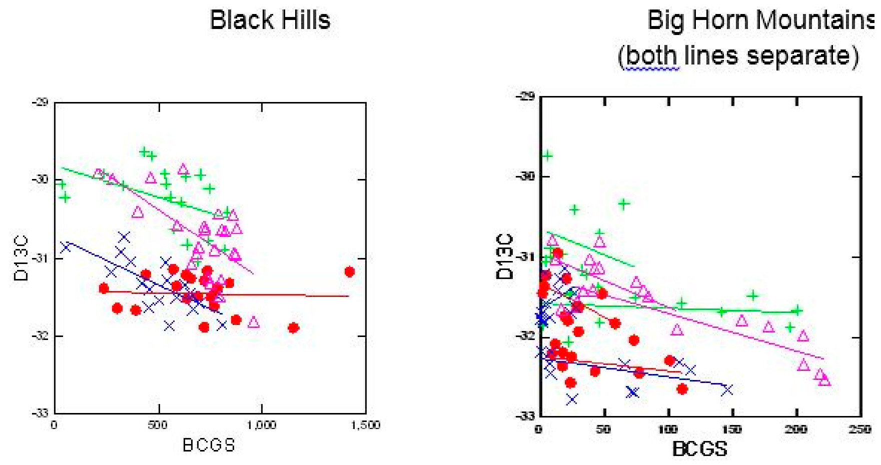

(**A**)

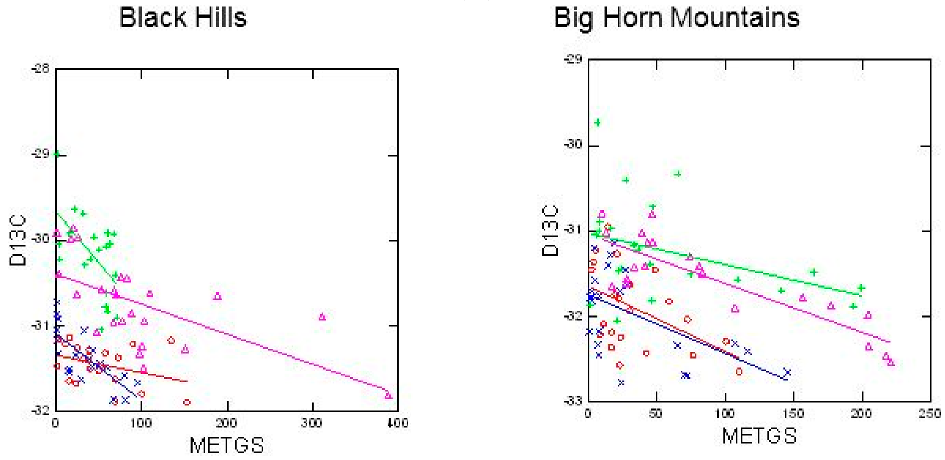

(**B**)

Figure S4. *Cont.*

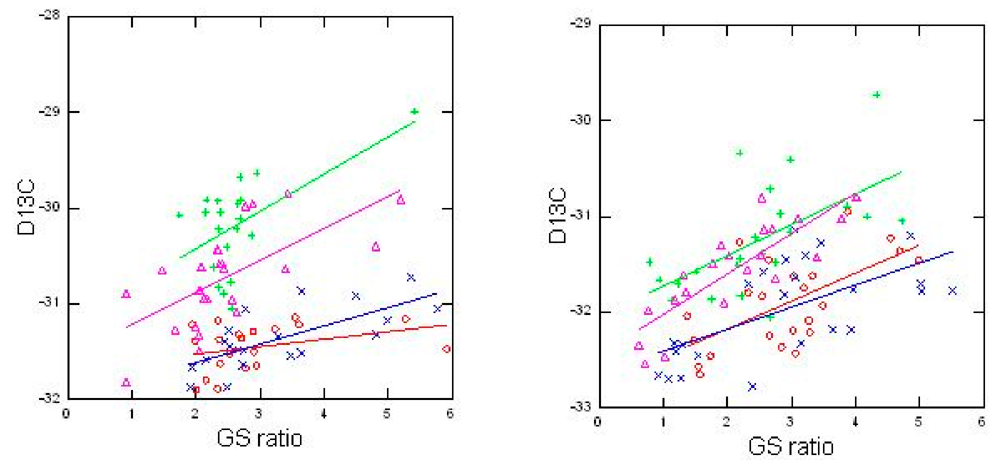

(**C**)

Figure S4. Correlations between carbon isotope ratio (D13C) and (**A**) branch chain glucosinolate concentration (μmol/g of 2-hydroxyl-1-methylethyl and 1-methylethyl glucosinolates); or (**B**) 6-methylsulfinylhexyl glucosinolate concentration; or (**C**) BCGS/METGS ratio (GS ratio). The different symbols represent different combinations of drought (drought, control) and herbivore (present, absent) treatments. **Green** plus sign = drought, herbivore absent; **Pink** triangle = drought, herbivore present; **Red** circle = control, herbivore absent; **Blue** *x* = control, herbivore present. The red circles are filled to highlight the different pattern exhibited under control watering and the absence of herbivores in the Black Hills ((**A**) only)). Statistical analysis in Table 7.

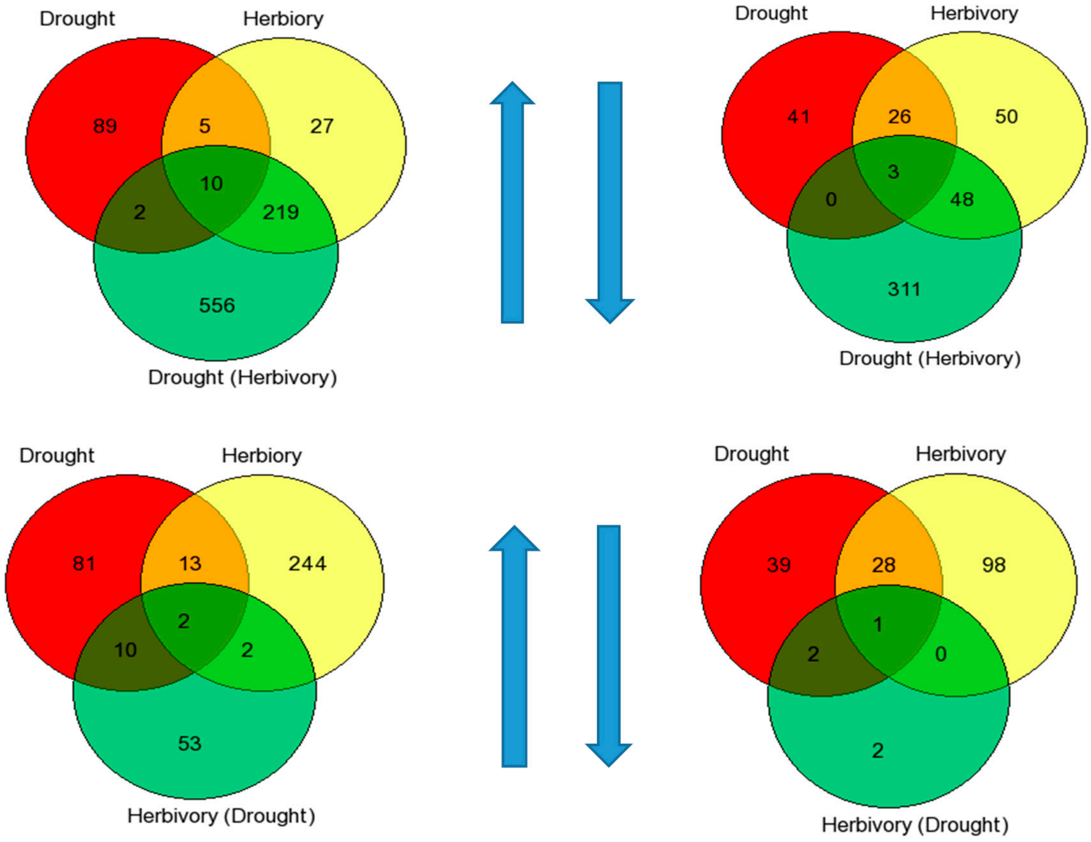

Figure S5. Venn diagrams for number of significantly up and down regulated genes. Red circle is Drought *vs.* control (no stress) and Yellow circle = Herbivory *vs.* control (no stress). Green circle in the top row of Venn diagrams is Herbivory *vs.* Control, both on drought-treated plants. Green circle in the bottom row of Venn diagrams is Drought *vs.* Control, both on plants fed upon by herbivores.

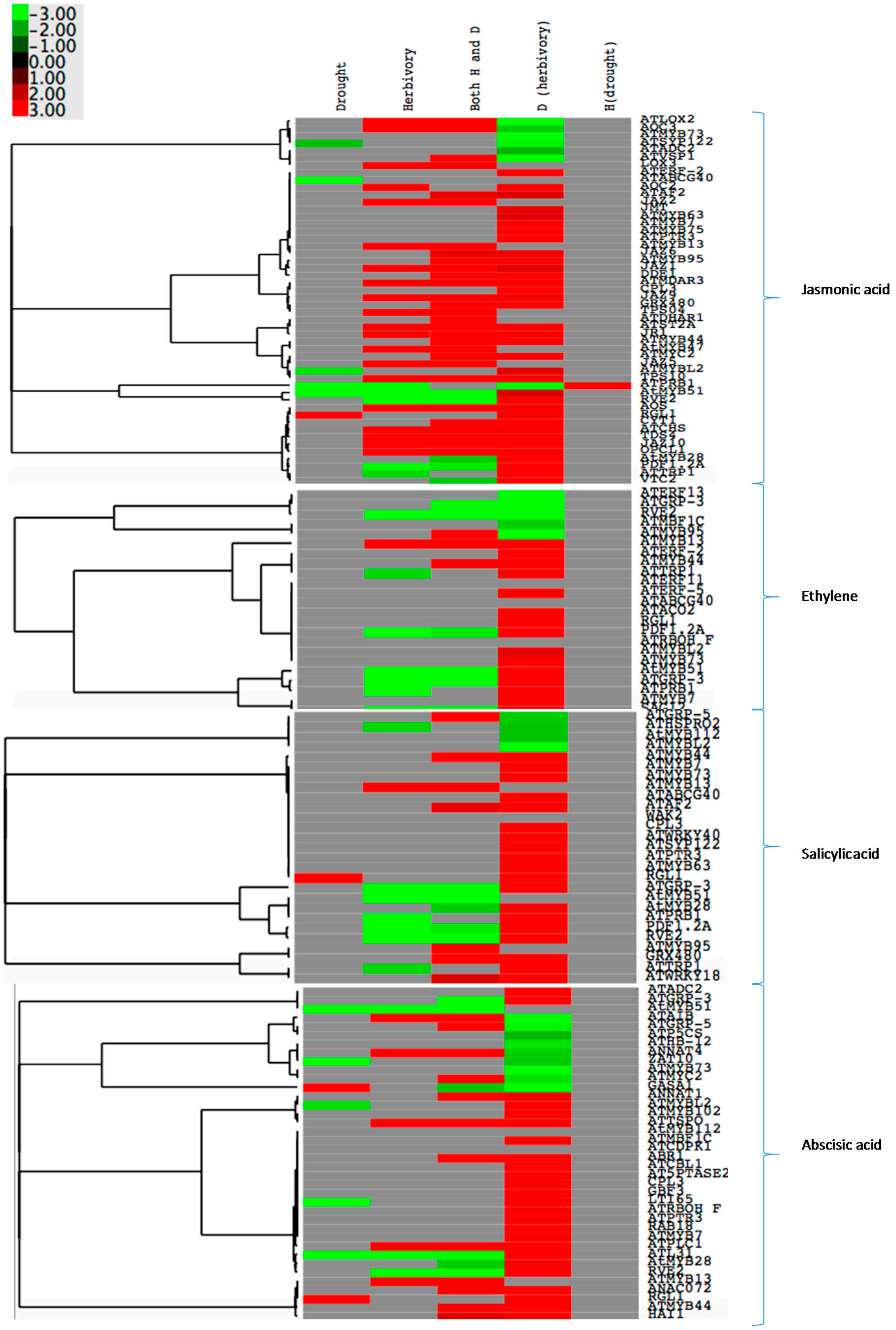

-3.00
-2.00
-1.00
0.00
1.00
2.00
3.00
Drought
Herbivory
Both H and D
D (herbivory)
H(drought)
Jasmonic acid
Ethylene
Salicylicacid
Abscisic acid

(**A**)

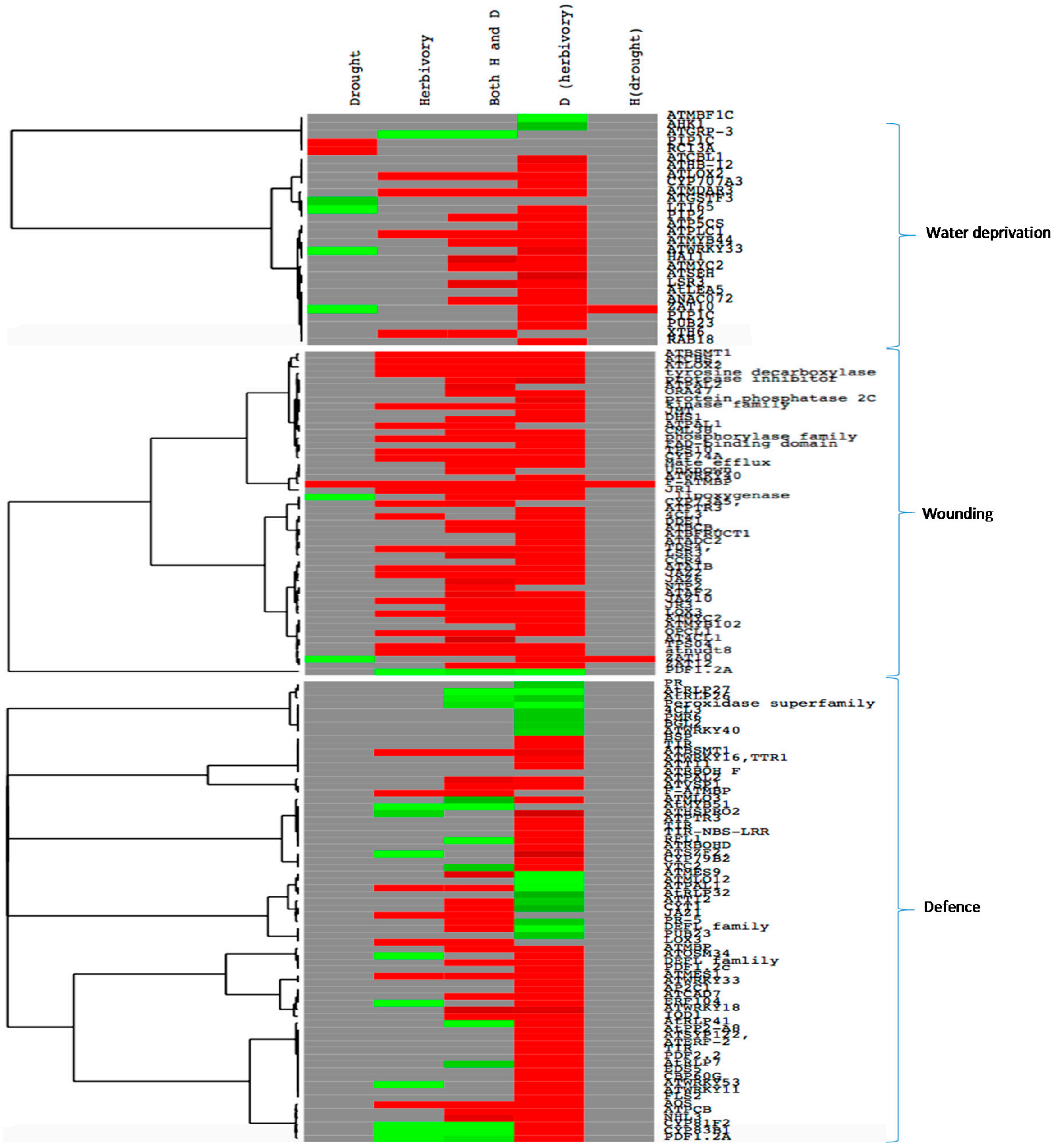

(**B**)

Figure S6. Gene expression hierarchical cluster analysis for signaling pathways (**A**) and water deprivation, wounding and defense (**B**) Effect of herbivory compared to controls without herbivory on drought treated plants is represented by "D (herbivory)." Likewise, drought treatments on plants fed upon represented by "H (drought)." Green and red represent significant down and up regulation (fold change) respectively (see legend for quantification of fold change by color variation). Also see Table S1 for fold change values.
